# Elevated Humoral Immune Response to SARS-CoV-2 at High Altitudes Revealed by an Anti-RBD “*In-House*” ELISA

**DOI:** 10.3389/fmed.2021.720988

**Published:** 2021-10-14

**Authors:** Rodrigo Hernán Tomas-Grau, Diego Ploper, César Luis Ávila, Esteban Vera Pingitore, Carolina Maldonado Galdeano, Silvina Chaves, Sergio Benjamín Socias, Agustín Stagnetto, Silvia Adriana Navarro, Rossana Elena Chahla, Mónica Aguilar López, Conrado Juan Llapur, Patricia Aznar, María Elena Alcorta, Dardo Costas, Isolina Flores, Dar Heinze, Gabriela Apfelbaum, Raul Mostoslavsky, Gustavo Mostoslavsky, Silvia Inés Cazorla, Gabriela del Valle Perdigón, Rosana Chehín

**Affiliations:** ^1^Instituto de Medicina Molecular y Celular Aplicada, Universidad Nacional de Tucumán-Consejo Nacional de Investigación Científicas y Técnicas- Sistema Provincial de Salud (UNT-CONICET-SIPROSA), Tucumán, Argentina; ^2^Centro de Referencia para Lactobacilos, Consejo Nacional de Investigación Científicas y Técnicas (CONICET), Tucumán, Argentina; ^3^Ministerio de Salud Pública de Tucumán, Tucumán, Argentina; ^4^Laboratorio de Salud Pública, Sistema Provincial de Salud, Hospital Néstor Kirchner, Tucumán, Argentina; ^5^Departamento de Residencias, Dirección General de Recursos Humanos (DGRRHH), Ministerio de Salud, Tucumán, Argentina; ^6^Section of Gastroenterology, Department of Medicine, Center for Regenerative Medicine, Boston University School of Medicine, Boston, MA, United States; ^7^Facultad de Medicina, Universidad Nacional de Tucumán, Tucumán, Argentina; ^8^The Massachusetts General Hospital Cancer Center, Harvard Medical School, Boston, MA, United States

**Keywords:** COVID-19, high altitude, SARS-CoV-2, RBD, ELISA

## Abstract

The severe acute respiratory syndrome coronavirus-2 (SARS-CoV-2) has caused a global pandemic with dramatic health and socioeconomic consequences. The Coronavirus Disease 2019 (COVID-19) challenges health systems to quickly respond by developing new diagnostic strategies that contribute to identify infected individuals, monitor infections, perform contact-tracing, and limit the spread of the virus. In this brief report, we developed a highly sensitive, specific, and precise “*In-House*” ELISA to correctly discriminate previously SARS-CoV-2-infected and non-infected individuals and study population seroprevalence. Among 758 individuals evaluated for anti-SARS-CoV-2 serology in the province of Tucumán, Argentina, we found a weak correlation between antibodies elicited against the RBD, the receptor-binding domain of the Spike protein, and the nucleocapsid (N) antigens of this virus. Additionally, we detected mild levels of anti-RBD IgG antibodies in 33.6% of individuals diagnosed with COVID-19, while only 19% showed sufficient antibody titers to be considered as plasma donors. No differences in IgG anti-RBD titers were found between women and men, neither in between different age groups ranging from 18 to 60. Surprisingly, individuals from a high altitude village displayed elevated and longer lasting anti-RBD titers compared to those from a lower altitude city. To our knowledge, this is the first report correlating altitude with increased humoral immune response against SARS-CoV-2 infection.

## Introduction

The severe acute respiratory syndrome coronavirus-2 (SARS-CoV-2) pandemic has disrupted the worldwide supply chain for many diagnostic equipment and their components, challenging government agencies and private companies in their efforts to acquire reagents, testing kits, vaccines, and any Coronavirus Disease 2019 (COVID-19)-related technologies. Serological surveillance of anti-SARS-CoV-2 antibodies in the population provides a crucial tool for designing public health guidelines (1, 2). This is only possible if serological tests are sufficiently trustworthy, which basically implies the correct election of the technique, the target antigen, and the antibodies to be studied. The unavailability of a sensitive, robust, and cost-effective diagnostic kit in our region motivated us to develop an “*In-House*” ELISA for detecting antibodies against the receptor binding domain (RBD) of the Spike glycoprotein from SARS-CoV-2, which show a strong correlation with virus neutralization (3, 4), in the convalescent population of Tucumán-Argentina. Our test can potentially help shape local health policies, select appropriate convalescent plasma donors, diagnose patients with radiological COVID-19-compatible imaging but who tested PCR negative, and supervise the effectiveness of vaccines. This brief communication reports the development of an “*In-House*” ELISA that is being used to survey seroprevalence in our region, and has uncovered that inhabitants of Tafí del Valle, a village situated in the Andean foothills at more than 2,000 meters above median sea level (mamsl), showed significantly higher anti-RBD titers than the population of San Miguel de Tucumán, located around 400 mamsl. To our knowledge, this is the first study showing an increased antibody response against SARS-CoV-2 in high-altitude individuals, adding more evidence regarding the effect of altitude on the interplay between this novel coronavirus, the immune system and the outcome of the infection (5–10).

## Methods

### Recombinant SARS-CoV-2 S RBD-His Expression

A plasmid encoding a secreted his-tagged SARS-CoV-2 S RBD was obtained as a generous gift from Jared Feldman and Aaron G. Schmidt (Harvard University). The RBD sequence was PCR amplified and subcloned into a pHAGE2-EF1a-IRES-ZsGreen-W lentiviral vector (11). Lentiviral particles were produced by cotransfecting the lentiviral backbone together with appropriate packaging vectors into HEK293 cells at 70% confluency in a 100 mm petri dish. HEK293 were grown in high glucose medium supplemented with 10% fetal bovine serum and antibiotic/antimycotic. PEI 87 kDa (PolyAr87) was used as the transfection reagent. Culture media containing lentiviral particles was harvested after 24/48 h and used to transduce a fresh HEK293 culture at 70% confluency in one well of a 6-well plate rendering the transgenic cell line HEK293-SARS-CoV-2-RBD. Media was washed after 24 h and transduced cells were grown and expanded. Transduction efficiency was assessed by imaging the fluorescence of ZSGreen, expressed from the pHAGE2 lentiviral vector. For RBD-His production, HEK293-SARS-CoV-2-RBD cells were plated at 20% confluency in T175 flasks with high glucose medium supplemented with 10% fetal bovine serum plus antibiotic/antimycotic, and media containing secreted RBD-His was collected at after 48 and 96 h for further purification.

### Recombinant SARS-CoV-2 S RBD-His purification

Purification of RBD-His contained in the conditioned media was carried out by affinity chromatography using a 5 ml HisTrap HP column (GE Healthcare, UK) coupled to an Akta Pure 20 l (GE) chromatograph (FPLC). Briefly, the supernatant was harvested from the cell culture, supplemented with 20 mM imidazole, and filtered through a 0.22 μm pore size PVDF filter. The affinity column was equilibrated with 10 column volumes of buffer A (20 mM NaH_2_PO_4_, NaCl 500 mM, pH 7.4, 20 mM imidazole), loaded with 100 ml sample using a sample pump, and washed with 10 column volumes of buffer A at a flow rate of 4 ml/min. Bound proteins were eluted with a step gradient with five column volume of either 20, 50, and 100% of buffer B (20 mM NaH_2_PO_4_, NaCl 500 mM, pH 7.4, 500 mM imidazole). Fractions of 5 ml were collected and further analyzed using SDS-PAGE. Fractions with molecular weight compatible with the His-tagged RBD were pooled and dialyzed against 200 mM NaH_2_PO_4_, pH 6.5. The production yield for RBD was around 0.5 mg/100 ml of cell culture supernatant, with a purity level higher than 95%. The secreted RBD-His domain, efficiently purified by affinity chromatography, migrated as expected on an SDS-PAGE gel according to previous reports (12).

### ELISA Anti-RBD Antibody Binding Assay

In order to assemble an indirect ELISA test for the determination of anti-RBD IgG antibodies, flat polystyrene bottom plates (High Binding, Half-Area, Greiner 675061) were sensitized with 0.1 μg per well in PBS of the RBD for 18 h at 4°C. Blocking was performed with 10% Fetal Bovine Serum in PBS 10 mM pH 7.4 during 1 h at 37°C. Plates were then washed three times with 0.1% Tween in PBS. Sera were assayed at serial dilutions in 10% Fetal Bovine Serum in PBS of 1/100 and incubated for 1 h at 37°C. Peroxidase-conjugated immunoglobulins to human IgG (whole molecule) (Sigma A8667), diluted 1/35,000, were used as the secondary antibody. Plates were developed by adding TMB (3,3′,5,5′–Tetramethylbenzidine; BD OptEIAtm), incubated for 15 min in the dark, and the reaction was terminated using 4N H_2_SO_4_. Optical density (OD) was read by an ELISA reader (TECAN Spark) at 450 nm. Cutoff values were calculated using receiver operating characteristic curve (ROC). Titers were calculated as the dilution in which the optical density obtained was equal to the cutoff.

### Populations Studied

Serum samples (758) from volunteers recovered from oligosymptomatic infection with SARS-CoV-2, without requiring hospitalization, or close contacts of these, were provided by the Laboratory of Public Health of Tucumán (SIPROSA), Argentina. Individuals for this study were between 18 and 60 years old, presented good general health, and had not been vaccinated against SARS-CoV-2 at the time of the study. Serum samples were collected at least 3 weeks after COVID-19 diagnosis. All procedures were approved by the Bioethics Committee of the SIPROSA N° 29/2020. As negative controls, 26 sera obtained before December 2019 (pre-pandemic) were analyzed.

### Ethics Statement

All procedures were approved by the Bioethics Committee of the SIPROSA N° 29/2020. We obtained a written signed informed consent from each individual enrolled in the study.

The protocol was approved by the Research Department and Ethics Committee (SIPROSA-Tucumán Health Ministry). Identification: Dictamen CEI 29-2020. Contact: +54-9-0381-4526585-ext:120. See information at:

https://msptucuman.gov.ar/wordpress/wp-content/uploads/2021/03/FCI-SeroconversionRCSfinal.pdf.

https://msptucuman.gov.ar/wordpress/wp-content/uploads/2021/03/03.Serocoversion-DICTAMEN-19-1-2021.pdf.

### Statistical Analyses

The cutoff point for optimal sensitivity and specificity, as well as the other statistical parameters, was determined using the receiver operating characteristic (ROC) curve analysis applying the XL-STAT statistical software/program (Excel). Anti-RBD IgG titers, correlation and differences between means, were carried out in Prisma8.0 (GraphPad, San Diego, CA). For non-parametric variables, data were analyzed by Kruskal-Wallis or Kolmogorov–Smirnov test. Significant differences between groups are shown with the corresponding *P*-values. Significant differences are indicated with asterisks.

## Results

**Figure 1:**
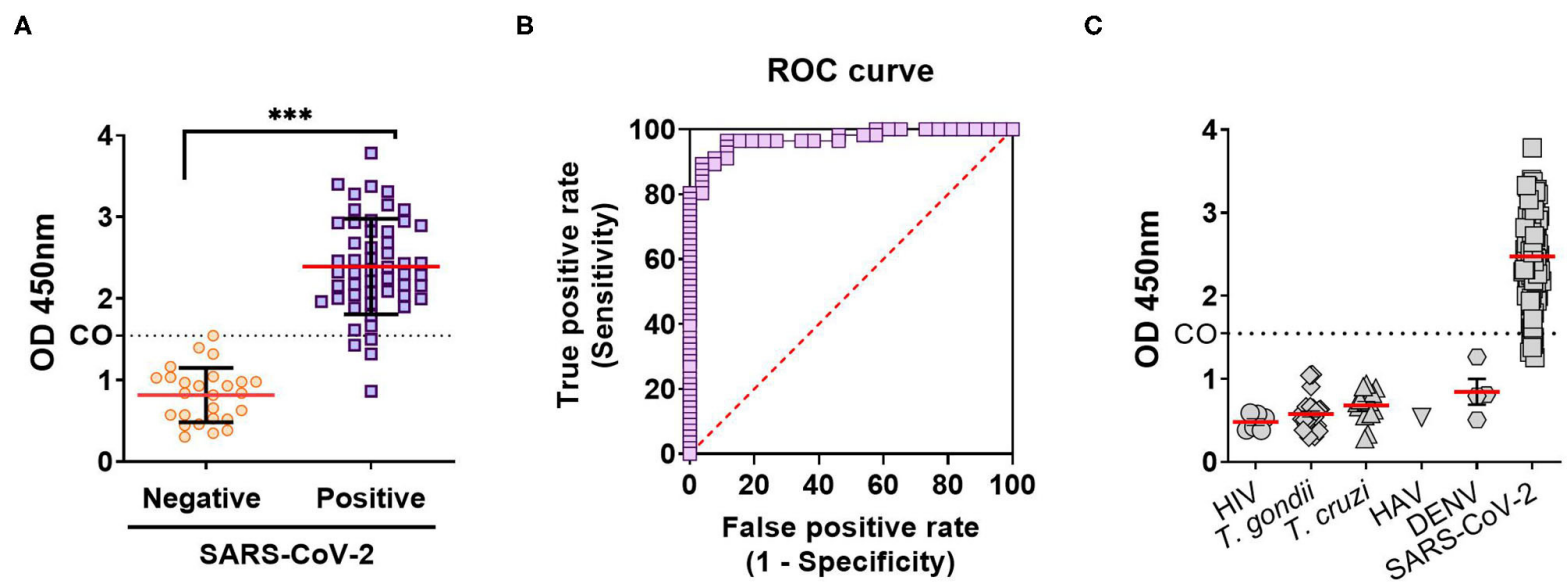
Diagnostic performance of an anti-RBD “*In-House*” ELISA for SARS-CoV-2. **(A)** Anti-RBD IgG antibodies on true negative (pre-pandemic sera, taken before December 2019) and true positive (by RT-PCR and CMIA anti-N IgG antigen test) samples for SARS-CoV-2 infection. Results are expressed as the OD 450 nm, and the cutoff (CO) calculated using the ROC curve (***p < 0.0001). **(B)** Diagnostic efficacy of the RBD antigens in SARS-CoV-2 infection calculated from ROC curve. **(C)** IgG antibodies against RBD in sera from individuals with infections by: HIV, human immunodeficiency virus; *T. gondii, Toxoplasma gondii*; *T. cruzi, Trypanosoma cruzi*; HAV, human hepatitis A virus; and DENV, Dengue virus.

**Figure 2:**
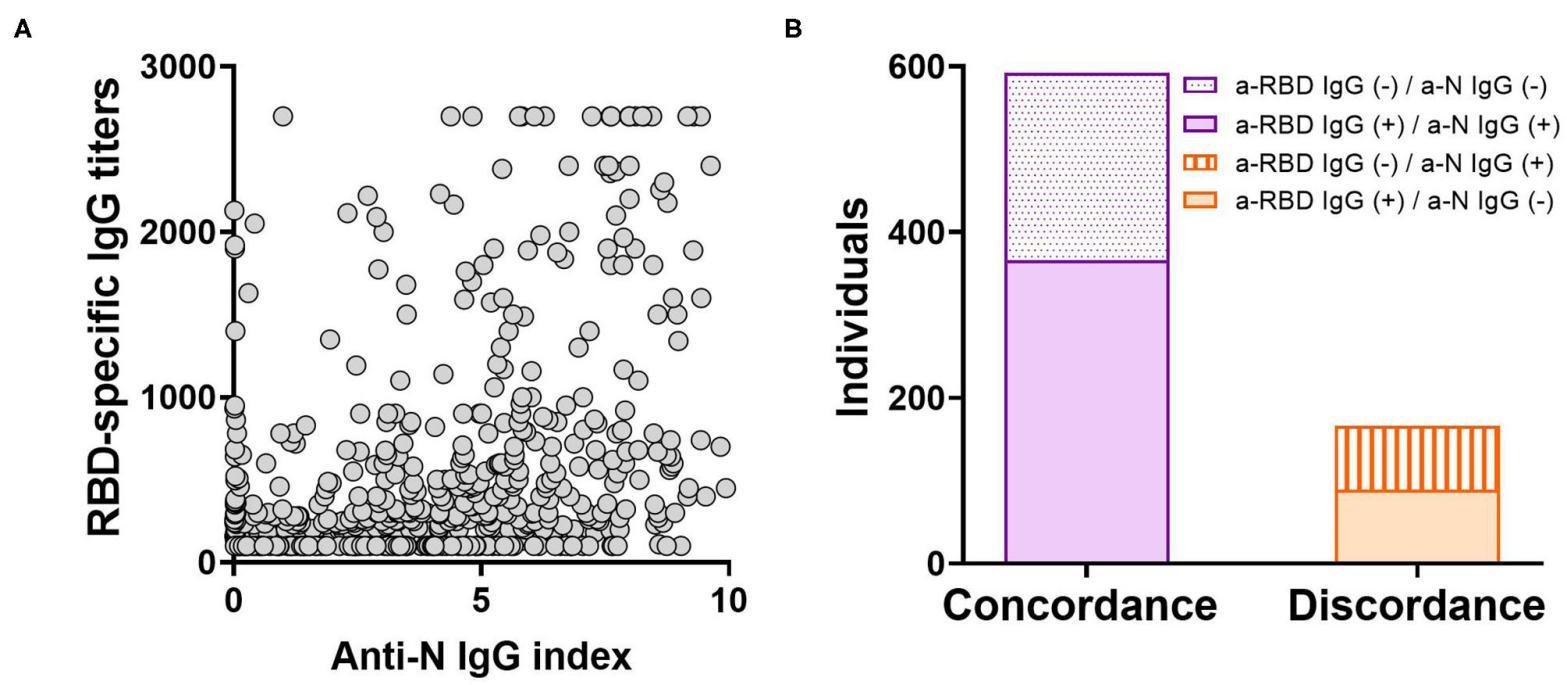
Comparison between the “*In-House*” ELISA and others tests in correctly discriminating previous infection by SARS-CoV-2. **(A)** Scatterplot depicting the relationship between titers from the anti-RBD “*In-House*” ELISA and the anti-N CMIA index for 758 individuals (*r* = 0.5048; *p* < 0.0001). The correlation was analyzed using Pearson Correlation Coefficient. **(B)** Concordance or discordance in results from the anti-RBD ELISA and the anti-N CMIA assay in the screening of IgG antibodies elicited after SARS-CoV-2 infection.

In order to evaluate anti-SARS-CoV-2 IgG antibody titers in our local population, an “*In-House*” ELISA assay was developed. For this, a transgenic HEK293 cell line that expresses and secretes RBD-His was produced by lentiviral transduction (Supplementary Figure 1). This ELISA was used to determine anti-RBD IgG titers from convalescent individuals of the local state health system (SIPROSA-Tucumán, Argentina). As positive controls, we analyzed 52 samples that tested SARS-CoV-2-positive by RT-PCR and for antibodies directed against the N protein by a commercially available chemiluminescent microparticle immunoassay (CMIA-Architect, Abbot). The anti-RBD ELISA correctly detected significantly higher anti-RBD titers from these samples compared to pre-pandemic sera (Figure 1A). Performance parameters showed a high degree of accuracy (AUC 0.988; 95% confidence interval 0.9778–1.000), sensitivity (92.2%), specificity (100%) and predictive value (1.00) (Figure 1B, Table 1). Antibodies elicited by other microorganisms did not interfere with the test (Figure 1C). Next, the “*In-House*” ELISA and the previously mentioned CMIA kit were compared. Among 595 individuals that were classified as SARS-CoV-2-positive by RT-PCR, plus 163 close contacts of these, the ELISA and CMIA displayed similar diagnostic abilities (Table 2). Although the correlation between titers of anti-RBD and the CMIA index was weak (*r* = 0.5048) (Figure 2A), a high concordance for presence or absence of both antibodies was observed (Figure 2B).

**Table 1:** Statistic parameters of the “*In-House*” ELISA test developed.

**ELISA for determination of RBD-specific IgG**	
**Statistic**	**Values**
Sensitivity	0.923
Specificity	1.00
True positives	48
False positives	0
True negatives	25
False negatives	4
Positive predictive value	1.0
Negative predictive value	0.862
Precision	0.948

**Table 2:** Percentages of the population studied (*n* = 758), either diagnosed as SARS-CoV-2 positive by RT-PCR, or close contacts of these, that have detectable SARS-CoV-2 anti-RBD or anti-N antibodies as measured by the “*In-House*” ELISA or CMIA, respectively.

		**a-RBD IgG (+)**	**a-N IgG (+)**	**a-RBD IgG (+)/a-N IgG (+)**
758 individuals	PCR (+)	59%	58.3%	47.9%
	*n* = 595	*n* = 351	*n* = 347	*n* = 285
	Close contact	63.8 %	58.9%	49.7%
	*n* = 163	*n* = 104	*n* = 96	*n* = 81

**Figure 3:**
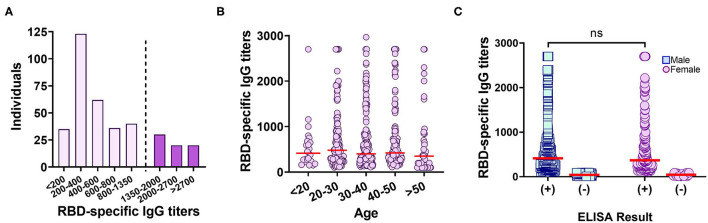
Anti-RBD IgG titers measured by an “*In-House*” ELISA in the studied population. **(A)** Distribution of anti-RBD titers among 366 true positive samples. Dotted line delimits the population that correlates with a probability ≥80% of having neutralizing titers ≥160 (13). **(B)** Distribution of anti-RBD titers segregated into age groups. Red line: median. **(C)** Distribution of anti-RBD titers, for each gender, measured by the anti-RBD “*In-House*” ELISA. Red line: median. ns, *p* = 0.4940, Kolmogorov–Smirnov test.

Subsequently, the distribution of anti-RBD IgG titers among 347 true positive samples (confirmed by both RT-PCR and CMIA) collected between September and December 2020 (weeks before The National Vaccination Program began) was examined with the “*In-House*” ELISA. A preponderance of titers between 300 and 800 (37%) were observed (Figure 3A). When the complete set of 758 samples were analyzed and segregated into age groups, no differences were observed (Figure 3B, Table 3). In addition, no significant difference was found in median anti-RBD titers between male and females (Figure 3C, Table 3), although the percentage of negativity was much higher in males (48.9%) than in females (9.7%) (Table 3).

**Table 3:** Demographic factors and statistical parameters of individuals included in this study.

**Demographic factors**	**Categories**		***n***	**Mean**	**SD**	**Median**	**Range (max–min)**	**95% CI**	***p*-value**
Age groups	<20		22	567.7	554.8	413.5	150–2,700	236–680	0.1564
	20–30		140	731.1	692.1	478	103–2,700	370–580	
	30–40		200	682.1	686.2	400	109–2,965	300–500	
	40–50		128	738.1	744.9	420	110–2,700	333–530	
	>50		41	640	759.6	350	100–2,700	190–600	
Gender	Positive anti-RBD	Male	278	703.2	698.03	414.5	100–2,700	350–497	0.494
		Female	177	710.4	729.8	370	100–2,700	470–300	
	Negative anti-RBD	Male	266	45.6	24.2	44	0–99	36–46	0.2728
		Female	19	45.8	30.9	50	0–99	15–78	

*Genders are further categorized into positive or negative for the presence of anti-RBD antibodies detected by the “In-House” anti-RBD ELISA*.

**Figure 4:**
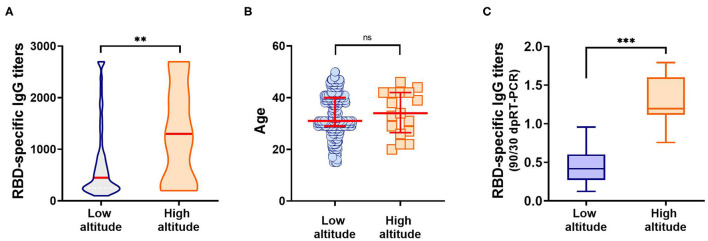
Anti-RBD IgG antibodies elicited in individuals from low (431 mamsl) and high altitudes (2,014 mamsl). **(A)** Specific IgG titers elicited at day 30 post-SARS-CoV-2 diagnosis, in each population. Red line: median. **p < 0.01, Kolmogorov–Smirnov test. **(B)** Age distribution among individuals from the low altitude and high altitude groups studied. No statistical difference was observed between the ages of the low altitude vs. high altitude groups when analyzed by the Kolmogorov–Smirnov test (*p* = 0.6277). Mean and standard deviation for each group are depicted in red. **(C)** Evolution of anti-RBD response against SARS-CoV-2 after 90 days post-diagnosis. Results represent the ratio between RBD-specific IgG titers at day 90 and day 30 post-diagnosis. ***p < 0.001, Kolmogorov–Smirnov test.

Surprisingly, we observed significantly higher anti-RBD titers in individuals from the high altitude village (2,014 mamsl) of Tafí del Valle (*n* = 17/3,403, 0.411% of the population) compared to titers from the lower altitude (431 mamsl) San Miguel de Tucumán (*n* = 574/1.448.188, 0.039% of the population) (Figure 4A). There was no statistical difference in age distribution between the high and low-altitude groups analyzed, underscoring that the difference observed in anti-RBD titers was not due to age differences between the groups (Figure 4B, Table 4). Interestingly, high altitude individuals sustained high specific antibody titers at day 90 post-COVID-19 diagnosis (Figure 4C, Table 4).

**Table 4:** Statistical parameters of the comparison between anti-RBD IgG antibodies elicited in individuals from low or high altitudes.

**Demographic factors**	**Categories**	***n***	**Mean**	**SD**	**Median**	**Range (max–min)**	**95% CI**	***p*-value**
RBD-specific IgG titer	Low altitude	574	727.5	712.5	450	100–2,600	384–497	0.0037^**^
	High altitude	17	1,284	930.2	1,300	200–2,500	260–1,965	
Age	Low altitude	494	33	8.07	31	15–35	31–38	0.627
	High altitude	17	34.06	8.56	34	20–26	27–42	
90/30 dpRT-PCR	Low altitude	18	0.4369	0.2179	0.4179	0.12–0.95	0.27–0.60	0.0002^***^
	High altitude	7	1.274	0.3385	1.194	0.76–1.79	0.76–1.79	

** *Significant difference p < 0.01*;

*** *significant difference p < 0.01 (Kolmogorov–Smirnov test)*.

## Discussion

The new coronavirus (SARS-CoV-2) infection has reached every continent, with new variants spreading quickly. Among patients infected with SARS-CoV-2, the progression of disease is highly variable (14, 15). SARS-CoV-2 pathogenicity results from an acute excessive viral replication followed by an uncontrolled inflammation and an exacerbated immunity. As the virus replicates, the adaptive immunity is stimulated to generate cellular and humoral responses in order to control the infection.

The role of sensitive molecular diagnostic techniques, such as RT-PCR and rapid antigen tests, are essential for the diagnosis of SARS-CoV-2 infection. Nevertheless, immunoserological tests have evolved as an indispensable tool, for example, in screening potential plasma donors with high titers of anti-SARS-CoV-2 neutralizing antibodies, given the reported success of convalescent plasma therapy for COVID-19 when administered at early stages of the disease (16). Many approaches have demonstrated that protection against SARS-CoV-2 is positively correlated with the development of high titers of neutralizing antibodies (3, 17–19). Due to its role in viral entry into the host cell, the RBD of S emerged as a potential target antigen for the development of preventive and therapeutic strategies against COVID-19 (3, 13, 20–23). Equally as important is the usefulness of RBD for the diagnosis of SARS-CoV-2 infection, as supported by epidemiological data and molecular diagnosis (24, 25).

By creating a stable cell line expressing high levels of RBD-His we were able to ensure a sufficient amount of antigen that enabled us to test to thousands of patients, reflected in this and other ongoing studies. The immobilization of this antigen to ELISA plates allowed for the assembly of a highly sensitive and specific ELISA assay that showed an AUC of 0.988 for the detection of anti-RBD IgG elicited after SARS-CoV-2 infection (Figures 1A–C). According to the traditional academic point system, an AUC between 0.90 and 1.0 indicates the antigen is an excellent ligand to correctly discriminate between the two groups (infected and non-infected) (26).

Importantly, the ability of the “*In-House*” ELISA to correctly discriminate the occurrence or not of a SARS-CoV-2 infection was compared with other molecular and serological tests. We observed that RBD specific IgG antibodies were elicited in 59% of individuals previously diagnosed as SARS-CoV-2 positive. However, only 19% of individuals presented titers equal to or above 1.350, an amount shown to have above 80% probability of possessing virus neutralization titers above 160, the FDA-recommended level for convalescent plasma use for treating COVID-19 (13) (Figure 3A). Our data showed that one out of five plasmas from COVID-19 recovered individuals were suitable candidates as donor for convalescent plasma therapy.

It has been reported that following infection, antibodies directed against RBD and N antigens begin to be detectable at slightly different times and in different amounts (27, 28). Therefore, high levels of antibodies elicited against one antigen do not imply the presence of similar amounts of the other (Figure 2). Our “*In-House*” ELISA test showed high concordance with the commercial CMIA Architect by Abbott in discriminating presence or absence of IgG antibodies against SARS-CoV-2. We also found that only 9.7% out of 196 female individuals tested negative for anti-RBD IgG, compared to 48.9% in the case of males. This is in accordance with the fact that female sex is associated with greater SARS-CoV-2 antibody levels in disease early phase (29). However, the average titers induced by each sex group showed no statistical difference (Figure 3C). The distribution of RBD-specific IgG antibodies in the population of Tucumán was consistent with previous seroprevalence reports (25).

Among the population studied, we noted that a high proportion of individuals that reside in high altitude villages showed high anti-RBD IgG antibodies. Further analysis confirmed that individuals from Tafí del Valle, a mountain village situated at 2014 mamsl, presented increased and long lasting antibodies against RBD compared to individuals from San Miguel de Tucumán, located at 431 mamsl (Figure 4). High altitude may play, at least in part, an important role in triggering a high and long-lasting humoral immune response of SARS-CoV-2, and might help explain previous publications reporting altitude as a protective factor for COVID-19 (5, 6, 10, 30). In the group of study, residents from Tafí del Valle presented an asymptomatic or very mild COVID-19, with none of them requiring hospitalization. This interesting finding represents a starting-point for future studies in the province of Tucumán to establish causality of environmental conditions with SARS-CoV-2 infection, and contribute to the implementation of policies to prevent and control the local spread of COVID-19.

Still, the effect of altitude in COVID-19 remains unclear. Recent epidemiological data have been used to propose that altitude of residence may not only influence those environmental features considered key to lesser viral transmission, but also susceptibility to more severe forms of COVID-19 through hypoxic-hypobaria driven genomic or non-genomic adaptations specific to high-altitude populations (30–32). Accordingly, Arias-Reyes et al. (5, 7), have reported a lower absolute number of COVID-19 cases at higher altitudes in Bolivia and Tibet. A similar protective effect was described in Bogotá by Cano-Pérez et al. (33). Segovia-Juarez et al. (34), by collecting data form provinces with altitudes ranging from 3 to 4,342 m, confirm that infection with COVID-19 at high altitude is reduced. However, case-fatality rate was not dependent on altitude. The authors also presented the first evidence that female protection toward death by COVID-19 is reduced as altitude of residence increases. A detailed comparison between the incidence, viral transmission, and severity of COVID-19 performed with data from 23 countries in the Americas has been recently published and also suggests a protective role of altitude (7).

In contrast, the high rate of SARS-CoV-2 seropositivity observed in La Rinconada, Perú, does not support a protective effect of high-altitude against COVID-19 spread, and demonstrates its large dissemination in vulnerable populations (35). Previously, Xi et al. (36), have also reported very few COVID-19 cases in the lowland countries Qinghai-Tibetan, China. Studies in the USA and Mexico have also shown that mortality due to COVID-19 was greater in cities with altitude higher than 2,000 m vs. those located below 1,500 m (37).

Finally, a publication from Italy showed no association of COVID-19 with altitude (38). The findings discussed above are clear evidences of the complex interplay between altitude, transmission, and mortality of SARS-COV-2 infection.

However, future studies with larger high-altitude populations should to shed light on the role of hypobaric hypoxia-adapted mechanism in SARS-COV-2 infection and humoral immune response. A compromised half-live of the virus caused by the high-altitude environment, a hypoxia mediated down regulation of angiotensin-converting enzyme 2 (ACE2) on the pulmonary epithelium, and/or immunomodulation mechanisms elicited, could help at least in part to understand this fine balance between the virus and the immune response to natural infection. In this context, our study provides an additional parameter, highly neutralizing anti-RBD antibodies, as a factor that may be influenced by altitude.

## Data Availability Statement

The raw data supporting the conclusions of this article will be made available by the authors, without undue reservation.

## Ethics Statement

The studies involving human participants were reviewed and approved by Comité de Etica-Dirección de Investigación-SIPROSA (Ministerio de Salud de Tucumán). The patients/participants provided their written informed consent to participate in this study.

## Author Contributions

RChe, RM, GM, GP, GA, and CL: conceptualization. RT-G, DP, CÁ, EV, CM, SCa, SS, AS, SN, and DH: methodology. MAL, PA, DC, IF, and MEA: investigation. RT-G, DP, GP, GA, GM, and RM: visualization. RCha, RChe, DP, SS, and CÁ: funding acquisition. RChe, SCa, EV, DC, and IF: supervision. DP, RChe, GP, RT-G, and SCa: writing. GM, RM, RChe, DP, RT-G, SCa, and EV: writing–review and editing. All authors contributed to the article and approved the submitted version.

## Funding

This work was supported by SkyBIO LLC, the School of Medicine of the National University of Tucumán (UNT), and Tucumán State Government. This work was also partially funded by the Argentinean Research Council-CONICET (PIP 722 and 806), Argentinean Research Agency (PICT-MINCYT3379, and PICT2018-02989), National University of Tucumán Grant (PIUNT-UNT D644/1 and D624). DP was supported by a Fundación Florencio Fiorini award.

## Conflict of Interest

This study received funding from SkyBIO LLC. The funder was not involved in the study design, collection, analysis, interpretation of data, the writing of this article or the decision to submit it for publication. The authors declare that the research was conducted in the absence of any commercial or financial relationships that could be construed as a potential conflict of interest.

## Publisher's Note

All claims expressed in this article are solely those of the authors and do not necessarily represent those of their affiliated organizations, or those of the publisher, the editors and the reviewers. Any product that may be evaluated in this article, or claim that may be made by its manufacturer, is not guaranteed or endorsed by the publisher.

## Supplementary Material

Supplementary Figure 1 Expression and purification of the recombinant RBD of the Spike protein from SARS-CoV-2. **(A–C)** Highly efficient transduction of HEK293 cells with pHAGE2 lentiviral particles coding for a secreted form of RBD-His can be observed by expression of the fluorescent reporter ZsGreen, co-expressed from the same construct. **(D)** Elution profile of the RBD-containing supernatant purified by HPLC using a HisTrap column. The purified RBD protein appeared in the second absorbance peak (blue) as indicated (black arrow). **(E)** Coomassie Brilliant Blue-stained SDS-PAGE gel showed that purified RBD-His migrated at the expected molecular weight (37 kDa) (lane 1); lane 2: molecular weight marker.

## Abbreviations

RBD: receptor binding domain
ROC: receiver operating characteristic curve
ELISA: enzyme linked immunosorbent assay
mamsl: meters above median sea level.
