# Supplementary figures and images for: Elevated Humoral Immune Response to SARS-CoV-2 at High Altitudes Revealed by an Anti-RBD “In-House” ELISA
Source: Front Med (Lausanne). 2021 Oct 14;8:720988. doi: 10.3389/fmed.2021.720988 (PMC8551828; doi:10.3389/fmed.2021.720988)

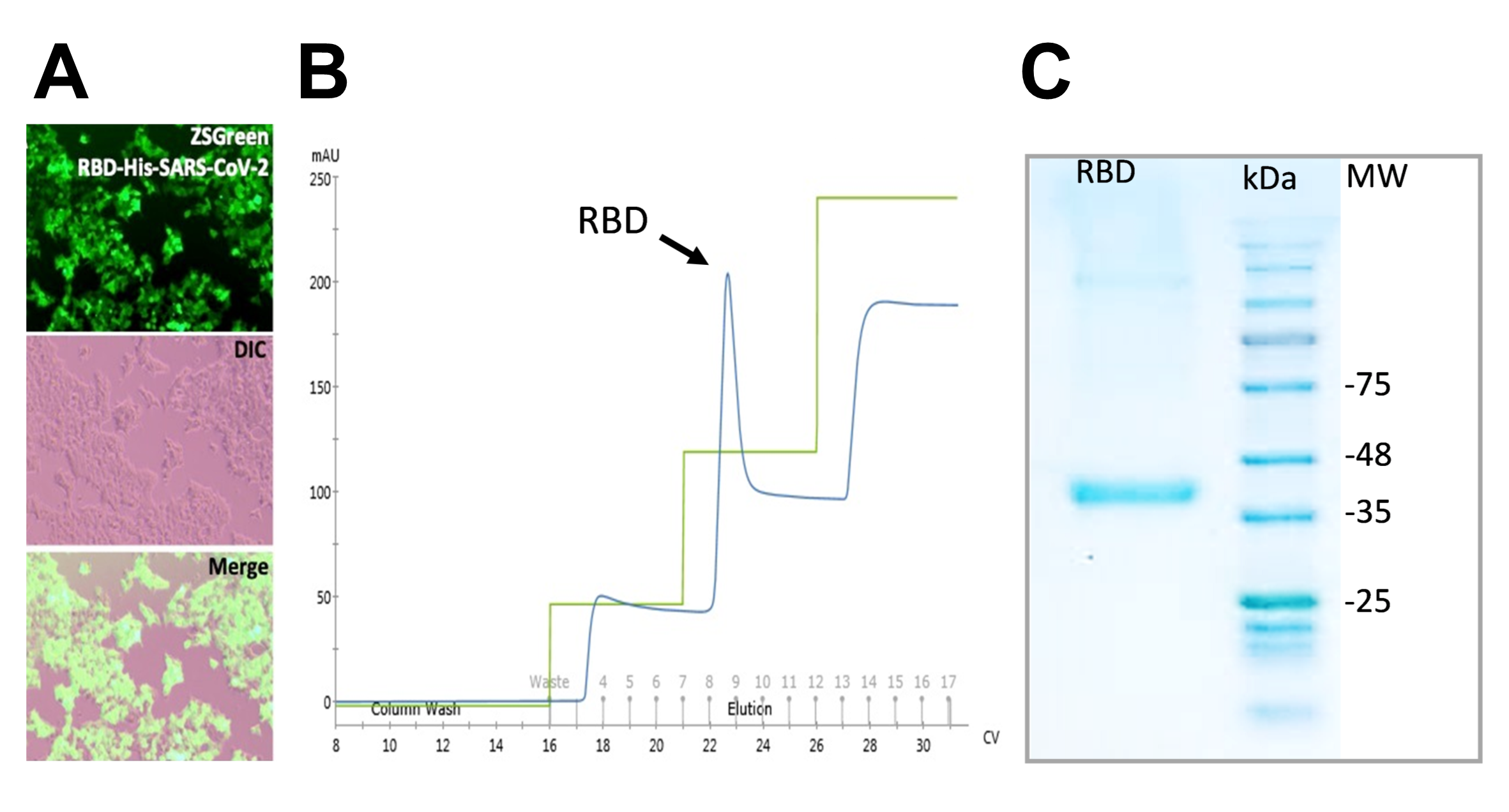

Supplement: Supplementary Figure 1 — Expression and purification of the recombinant RBD of the Spike protein from SARS-CoV-2. (A–C) Highly efficient transduction of HEK293 cells with pHAGE2 lentiviral particles coding for a secreted form of RBD-His can be observed by expression of the fluorescent reporter ZsGreen, co-expressed from the same construct. (D) Elution profile of the RBD-containing supernatant purified by HPLC using a HisTrap column. The purified RBD protein appeared in the second absorbance peak (blue) as indicated (black arrow). (E) Coomassie Brilliant Blue-stained SDS-PAGE gel showed that purified RBD-His migrated at the expected molecular weight (37 kDa) (lane 1); lane 2: molecular weight marker. [file Image_1.TIF]
